# Understanding the Cellular Function of TRPV2 Channel through Generation of Specific Monoclonal Antibodies

**DOI:** 10.1371/journal.pone.0085392

**Published:** 2013-12-31

**Authors:** Matthew R. Cohen, Kevin W. Huynh, Daniel Cawley, Vera Y. Moiseenkova-Bell

**Affiliations:** 1 Department of Physiology & Biophysics, School of Medicine, Case Western Reserve University, Cleveland, Ohio, United States of America; 2 Department of Pharmacology, School of Medicine, Case Western Reserve University, Cleveland, Ohio, United States of America; 3 Monoclonal Antibody Core Facility, Vaccine and Gene Therapy Institute, Oregon Health and Science University, Beaverton, Oregon, United States of America; St. Joseph’s Hospital and Medical Center, United States of America

## Abstract

Transient receptor potential vanilloid 2 (TRPV2) is a Ca^2+^-permeable nonselective cation channel proposed to play a critical role in a wide array of cellular processes. Although TRPV2 surface expression was originally determined to be sensitive to growth factor signaling, regulated trafficking of TRPV2 has remained controversial. TRPV2 has proven difficult to study due to the lack of specific pharmacological tools to modulate channel activity; therefore, most studies of the cellular function of TRPV2 rely on immuno-detection techniques. Polyclonal antibodies against TRPV2 have not been properly validated and characterized, which may contribute to conflicting results regarding its function in the cell. Here, we developed monoclonal antibodies using full-length TRPV2 as an antigen. Extensive characterization of these antibodies and comparison to commonly used commercially available TRPV2 antibodies revealed that while monoclonal antibodies generated in our laboratory were suitable for detection of endogenous TRPV2 by western blot, immunoprecipitation and immunocytochemistry, the commercially available polyclonal antibodies we tested were not able to recognize endogenous TRPV2. We used our newly generated and validated TRPV2 antibodies to determine the effects of insulin-like growth factor 1 (IGF-1) on TRPV2 surface expression in heterologous and endogenous expression systems. We found that IGF-1 had little to no effect on trafficking and plasma membrane expression of TRPV2. Overall, these new TRPV2 monoclonal antibodies served to dispel the controversy of the effects of IGF-1 on TRPV2 plasma membrane expression and will clarify the role TRPV2 plays in cellular function. Furthermore, our strategy of using full-length tetrameric TRP channels may allow for the generation of antibodies against other TRP channels of unclear function.

## Introduction

The transient receptor potential (TRP) family of nonselective cation channels contains 28 recently identified mammalian homologs grouped into six subfamilies based on sequence homology: vanilloid (TRPV), canonical (TRPC), melastatin (TRPM), ankyrin (TRPA), mucolipin (TRPML), and polycystin (TRPP) [1]. TRP channels are proposed to function in a broad range of processes, although the exact cellular function of several TRP channels remains elusive. Considerable challenges in elucidating the function of TRP channels include the absence of the specific activators, inhibitors and antibodies for each individual family member [2]. The controversial function of TRPV subfamily members provides a good example of this current problem in TRP field.

The TRPV subfamily consists of six members (TRPV1–6) [1]. TRPV1 has been the most comprehensively studied TRP channel due to its role in noxious pain sensation [3]. Capsaicin, the active ingredient in chili peppers, is a specific activator of TRPV1 and was used for identification and characterization of the channel properties [4]. Specific activators and inhibitors, in addition to TRPV1 knockout mice, have consistently indicated that TRPV1 acts as a heat and pain sensor *in vivo* [5]. TRPV2 shares nearly 50% sequence identity with TRPV1 and was cloned concurrently by two laboratories [6], [7]. One group identified TRPV2 as an insulin-like growth factor-1 (IGF-1) sensitive Ca^2+^ channel. Upon exposure to IGF-1, heterologously expressed TRPV2 was shown to move from intracellular membranes to the cell surface, where it mediated Ca^2+^ influx [7]. However, later studies indicated that, while IGF-1 signaling may affect TRPV2 activity, it does not affect surface expression of the channel [8], [9]. TRPV2 was also originally shown to function as a noxious heat sensor in a heterologous expression system [6]. Later, TRPV2 was also proposed to function in osmo- and mechanosensation [10]. However, recently generated TRPV2 knockout mice display normal sensory transduction, suggesting that TRPV2 does not function as a noxious heat and mechanical sensor *in vivo* [11]. Additionally these mice were subject to perinatal lethality, indicating that TRPV2 has another, as yet unknown function [11].

The physiological function of endogenous TRPV2 has remained controversial due to the lack of pharmacological and biochemical tools to study this channel [12]. Unlike TRPV1, TRPV2 is not modulated by vanilloids, such as capsaicin [6]. Putative activators and inhibitors of TRPV2 such as 2-aminoethoxydiphenyl borate (2-APB) and SFK96365 affect other TRP channel family members and non-selective cation permeation pathways [13]. The only other tools for exploring the endogenous function of the channel have been commercially available polyclonal antibodies generated against small linear peptides derived from TRPV2. Based on these available tools, TRPV2 has been proposed to play a major functional role in diseases such as muscular dystrophy, cardiomyopathy, prostate cancer, bladder cancer, glioblastoma development and diabetes. [14], [15], [16], [17], [18]. Recently, TRPV2 has also been proposed to be involved in immune response mechanisms, neuronal development and insulin secretion [18], [19], [20]. However, these results have not been without controversy [12]. Differences in commercially available polyclonal antibodies utilized in many of these studies may be especially problematic for studying endogenously expressed TRPV2.

Since pharmacological effectors of TRPV2 are non-specific and endogenous TRPV2 ligands are unknown, efforts to understand the proposed endogenous function of TRPV2 have been hindered. Validation of currently available antibodies and generation of antibodies suitable for detection of endogenously expressed TRPV2 would provide the best path towards accelerating understanding the cellular function of this ion channel. Here we generated for the first time TRPV2 monoclonal antibodies raised against full-length, tetrametric TRPV2. These antibodies, together with commercially available TRPV2 polyclonal antibodies, were characterized for detection of recombinant and endogenously expressed TRPV2. We found that while monoclonal antibodies generated in our laboratory were suitable for detection of endogenously expressed TRPV2 by western blot and immunocytochemistry, commercially available antibodies raised against synthetic and recombinant linear peptides derived from TRPV2 failed to detect endogenously expressed TRPV2 in cell lines or rodent tissues. Importantly, we tested the effects of IGF-1 on endogenous TRPV2 translocation to the plasma membrane using our newly generated and validated monoclonal antibodies. We found that IGF-1 had little to no effect on surface expression of TRPV2, suggesting that TRPV2 predominantly localizes to intracellular membranes. We expect that these results will allow for further investigation of the role endogenously expressed TRPV2 plays in cell function and disease.

## Materials and Methods

### Ethics Statement

All animal studies were approved by the Case Western Reserve University Institutional Animal Care and Use Committee.

### Plasmids

The following cDNAs were kindly provided by the indicated investigators: rat TRPV2 ankryin repeat domain, Rachelle Gaudet (Harvard Medical School); rat TRPV2 C-terminus with MBP-tag, Sharona Gordon (University of Washington); rat TRPV1 and TRPV2, David Julius (University of California San Francisco).

### Protein Expression and Purification

Full-length 1D4-tagged rat TRPV2 was cloned into a YepM plasmid and overexpressed in BJ5457 S. cerevisiae (ATCC). TRPV2 membranes were prepared and solubilized in 0.087% wt/v lauryl maltose-neopentyl glycol (MNG) (Anatrace), 20 mM HEPES (pH 8.0), 150 mM NaCl, 5% glycerol, 1.0 mM DTT and 1 mM PMSF for one hour at 4°C. The insoluble fractions were pelleted by ultracentrifugation at 100,000 g, 4°C for 45 minutes. The soluble fraction containing TRPV2 protein was incubated overnight at 4°C with CNBr-activated Sepharose 4B coupled with 1D4 antibody. The column was packed and washed with washing buffer consisting of 0.006% decyl maltose-neopentyl glycol (Anatrace), 20 mM HEPES (pH 8.0), 150 mM NaCl, and 1 mM DTT. The TRPV2 protein was eluted with 3 mg/ml 1D4 peptide (Genescript USA) in washing buffer, concentrated and subjected to size-exclusion chromatography (SEC). Rat TRPV2 ankryin repeat domain (ARD) and MBP-tagged rat TRPV2 C-terminus were expressed in BL21 (DE3) cells and purified as previously reported [21], [22].

### TRPV2 Antibody Generation

Mouse monoclonal antibodies against full-length rat TRPV2 protein were obtained using standard methods [23]. TRPV2 antibodies were purified from hybridoma supernatants by protein G affinity chromatography.

### Commercially Available Antibodies

The following commercially available antibodies were used: α-VRL-1 SC-22520 and α-phospho-Akt (Santa Cruz), α-VRL-1 PC421 (Calbiochem), α-TRPV2 ACC-032 (Alomone Labs), α-Na,K-ATPase α1, α-pan-Akt and α-β-Actin (Cell Signaling).

### Cell Culture and Transfection

The following cell lines were kind gifts from the indicated investigators: HeLa cells, Marvin Nieman (Case Western Reserve University); F11 cells, Sharona Gordon (University of Washington) [21]. CHO-K1 cells were obtained from ATCC. Cells were maintained in a humidified atmosphere at 37°C and 5% CO_2_. HeLa cells were cultured in Dulbecco’s modified Eagle’s medium (DMEM) with high glucose (Invitrogen) supplemented with 10% FBS (Cellgro), 100 unit/mL penicillin and 100 µg/mL streptomycin (Invitrogen). F11 cells were cultured in F12 medium (Invitrogen) supplemented with 10% FBS, HAT supplement (Invitrogen), 100 unit/mL penicillin and 100 µg/mL streptomycin. CHO-K1 cells were cultured in F12 medium supplemented with 10% FBS, 100 unit/mL penicillin and 100 µg/mL streptomycin.

Transfection of plasmid was performed using polyethyleneimine (PEI; Polysciences) as a carrier. A solution containing serum-free DMEM, 20 mM HEPES pH 7.5, and 3 µg PEI per 1 µg plasmid was incubated for 10 min at room temperature to allow DNA:PEI complexes to form. The mixture was then added directly to growth medium overlaying the cells. After a 4-hour incubation period at 37°C, fresh growth medium was added to cells. Cells were harvested or fixed 16–24 hours after transfection.

TRPV2 siRNA (rat VRL-1 SMARTpool) and non-targeting siRNA were obtained from Dharmacon. Transfection of siRNA was performed using Lipofectamine 2000 (Invitrogen) as a carrier following the manufacturer’s protocol.

For protein extraction, cells were incubated in lysis buffer (150 mM NaCl, 2 mM EDTA, 50 mM Tris-HCl (pH 7.5), 1% Triton-X100 and a protease inhibitor cocktail) for 30 min on ice. Lysates were cleared at 20,000×g for 20 min. Protein concentrations were determined by BCA assay.

### Mouse Tissue Lysate Generation and Immunoprecipitation

Male mice (P24) were deeply anesthetized with isofluorane and decapitated. Brains and hearts were removed and homogenized by 10 strokes with a dounce homogenizer in lysis buffer. Homogenates were incubated on ice for 30 minutes and cleared by centrifugation at 20,000×g for 20 min and 100,000×g for 30 min to remove unbroken cells and insoluble membrane fragments. The supernatant was pre-cleared with 50 µl protein A/G agarose beads. 2.5 mg of pre-cleared lysate was incubated with 10 µg anti-TRPV2 antibody for 2 hours at 4°C. Next, 50 µl protein A/G agarose was added for 2 hours at 4°C to capture TRPV2 antibody complexes. Protein A/G agarose beads were washed 3 times in lysis buffer and proteins were eluted with Laemmli sample buffer. Samples were boiled at 95°C for 5 min. Immunoprecipitation of TRPV2 was analyzed by western blot with IR-dye-labeled TRPV2 2D6 antibody.

### Western Blot Analysis

Lysates and immunoprecipitates were loaded onto 10% Tris-Glycine gels (Invitrogen) and run at 135 V for 90 min. Proteins were transferred to nitrocelluolose at 40 V for 2.5 hours at 4°C. Protein transfer and equal loading was confirmed by Ponceau S staining. Membranes were blocked in TBS-T with 10% (w/v) non-fat dry milk powder and incubated with primary antibody for 1 hour at room temperature. Primary antibodies were detected with IR-dye-labeled secondary antibodies (LiCor; 0.1 µg/ml) using the Odyssey imaging system (LiCor). 2D6 and 17A11 were used at 1 µg/ml, TRPV2 ACC-032 (Alomone) at 2 µg/ml, VRL-1 PC421 (Calbiochem) at 5 µg/ml and VRL-1 SC-22520 (Santa Cruz) at 10 µg/ml.

### Immunocytochemistry

HeLa cells and CHO cells were seeded on glass coverslips in 24 well plates at a density of 1×10^4^ cells per well. The following day, cells were transfected with plasmid for 24 hours. Cells were washed in PBS, fixed in 4% paraformaldehyde and blocked and permeabilized in PBS containing 0.3% Triton-X100 (PBS-T) and 1.5% normal goat serum. For analysis of TRPV2 antibody recognition of TRPV2-1D4, TRPV2 monoclonal antibodies, cells were incubated in PBS-T containing TRPV2 antibody for 1 hour followed by Alexa 488-labeled goat anti-mouse for 1 hour. After 5 washes in PBS, cells were incubated with Alexa 568-labeled 1D4 antibody for 1 hour. For polyclonal TRPV2 antibodies, cells were incubated with TRPV2 antibody and 1D4 antibody for 1 hour followed by Alexa 488-labeled donkey anti-mouse and Alexa 594-labeled donkey anti-rabbit or donkey anti-goat. Coverslips were mounted onto glass slides using Vectashield mounting medium.

F11 cells were seeded in 35 mm iBidi dishes at a density of 3×10^4^ cells per dish. The following day, cells were washed in PBS containing 100 µM CaCl_2_, fixed in 4% paraformaldehyde and blocked and permeabilized in PBS-T containing 100 µM CaCl_2_ and 1.5% normal goat serum. After blocking and permeablilization, cells were incubated in primary antibody for 1 hour at room temperature. After 3 washes in PBS, cells were incubated in secondary antibody for 1 hour.

For IGF-1 experiments, CHO-K1 cells transiently expressing TRPV2 or F11 cells, which endogenously express TRPV2, were treated with serum-free medium for 6 hours. Vehicle or IGF-1 (20 ng/ml) was added to the cells at 37°C. Cells were then fixed and immunostained for TRPV2 with 17A11 antibody.

Images were obtained using a Leica TCS SP2 confocal microscope. Brightness and contrast of images were adjusted using Photoshop (Adobe). Only linear changes were applied.

2D6 and 17A11 were used at 2 µg/ml, TRPV2 ACC-032 (Alomone) at 4 µg/ml, VRL-1 PC421 (Calbiochem) at 10 µg/ml and VRL-1 SC-22520 (Santa Cruz) at 20 µg/ml.

### Biotinylation of Cell Surface Proteins

CHO-K1 cells transiently expressing TRPV2 and F11 cells were cultured to approximately 75% confluence in 10 cm dishes and serum-deprived for 6 hours. After the indicated treatments, cells were washed 3 times in PBS and biotinylation reagent (Sulfo-Link NHS-LC-biotin, Pierce, 0.5 mg/ml in PBS) was added at 37°C. Following a 30 min incubation period, the biotin reagent was removed and cells were washed twice in PBS containing 100 mM glycine and twice in PBS alone. Next, lysates were prepared as described previously and incubated with 50 µl of streptavidin-agarose beads (Invitrogen) for 1 hour at 4°C to capture biotinylated proteins. The beads were pelleted and washed 3 times in lysis buffer. Captured proteins were eluted with Laemmli sample buffer, boiled at 95°C for 5 min, and analyzed by western blot with indicated antibodies.

## Results

### Detection of Recombinant TRPV2 and Determination of TRPV2 Binding Region

**Figure 1:**
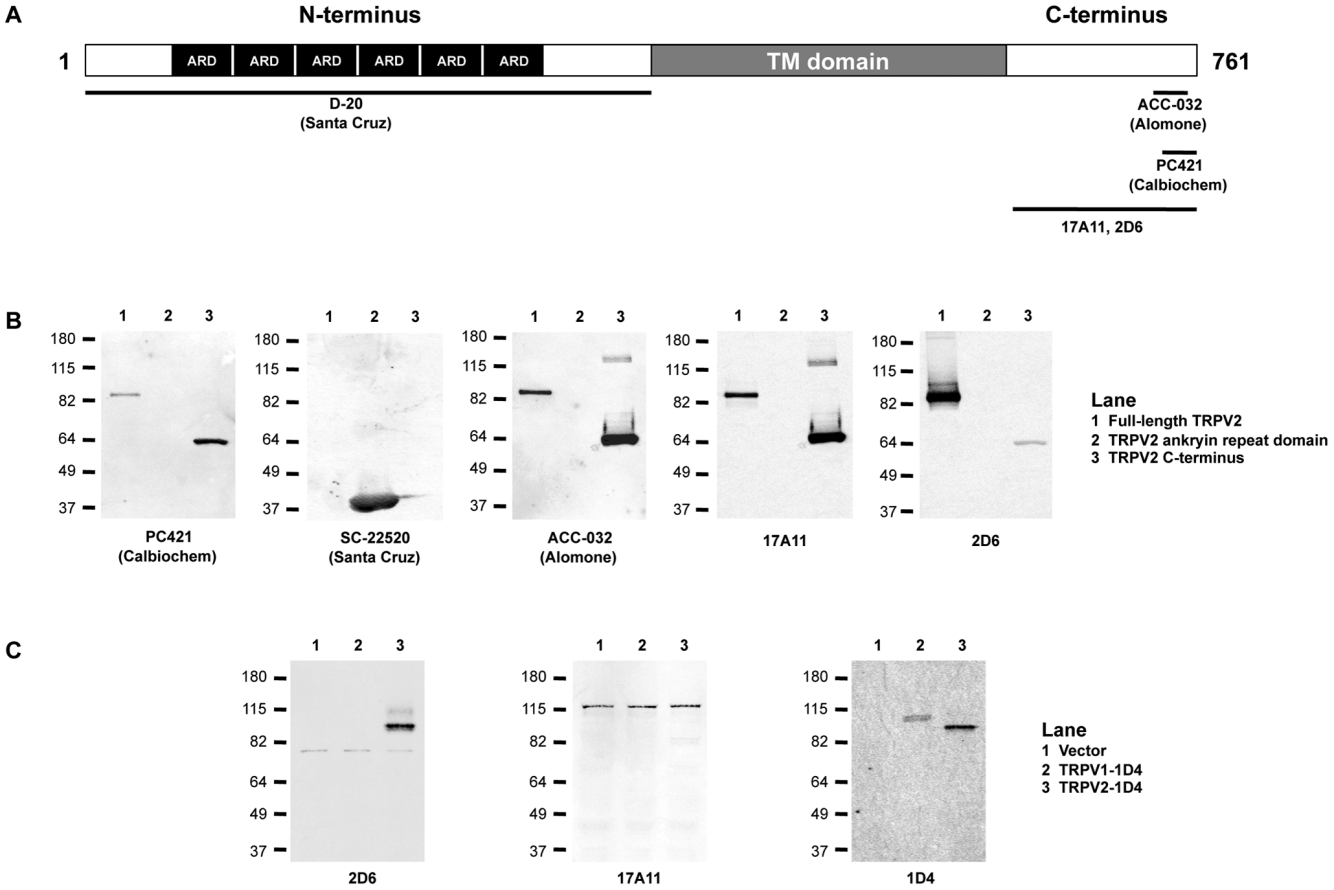
Detection of recombinant TRPV2 and mapping of the TRPV2 binding region. *A,* Schematic of the domain arrangement for a TRPV2 monomer with the approximate epitope sites for indicated TRPV2 antibodies. ARD, ankryin repeat domain; TM, transmembrane domain. *B,* Western blots with indicated TRPV2 antibodies against purified full-length rat TRPV2, purified rat TRPV2 ankryin repeat domain and purified rat TRPV2 C-terminus. **C,** Western blots with indicated TRPV2 antibodies against extracts from HeLa cells transiently transfected with empty vector, TRPV1-1D4 and TRPV2-1D4.

TRPV2 is an integral membrane protein that functions as a homotetramer [12]. Each monomer has an approximate molecular weight of 86 kDa and consists of 6 transmembrane spanning helices, 6 N-terminal ankryin repeats and a short C-terminus (Figure 1A) [12]. Divergence in the sequence between TRPV2 and other TRPV subfamily members resides in the distal C-terminus; therefore most available polyclonal TRPV2 antibodies were generated against synthetic or recombinant peptides derived from the C-terminus. The commercially available antibodies against C-terminal peptides from mouse and rat TRPV2 used in this study include PC421 (Calbiochem) and ACC-032 (Alomone). Additionally, a polyclonal antibody against an N-terminal peptide from human TRPV2 was tested (SC-22520; Santa Cruz). The antibodies generated in our laboratory were raised against full-length homotetrameric TRPV2 as an antigen.

To determine the specificity of these antibodies against TRPV2, western blots were performed against full-length recombinant rat TRPV2 as well as the soluble N-terminal ankryin repeat domain and C-terminus fused to maltose binding protein. Full-length recombinant TRPV2 was recognized by four of the five antibodies tested (Figure 1B). SC-22520 did not recognize full-length rat TRPV2. SC-22520 was raised against a peptide derived from human TRPV2 and is expected to have lower reactivity with rat TRPV2; it recognized the N-terminal ARD but not full-length TRPV2 (Figure 1B). As expected, PC421 and ACC-032, which were generated against a peptide from the distal C-terminus, recognized both recombinant full-length TRPV2 and the C-terminus (Figure 1B). Mouse monoclonal antibodies 2D6 and 17A11 generated in our laboratory also recognized full-length recombinant TRPV2 and the C-terminus (Figure 1B). The expected binding site of each antibody tested is mapped in the schematic in Figure 1A.

Since TRPV1 shares nearly 50% sequence identity with TRPV2, we tested our TRPV2 monoclonal antibodies for cross-reactivity with TRPV1 by heterologously overexpressing 1D4-tagged TRPV1 and TRPV2 in HeLa cells. 2D6 recognized TRPV2 but not TRPV1 in HeLa cells (Figure 1C). 17A11 showed very little immunoreactivity with TRPV2 expressed in HeLa cells and non-specific reactivity with a protein of approximately 120 kDa (Figure 1C). These results show that, of our monoclonal antibodies, 2D6 was the most suitable candidate for detection of endogenous TRPV2 by western blot.

### Recognition of Endogenously Expressed TRPV2

**Figure 2:**
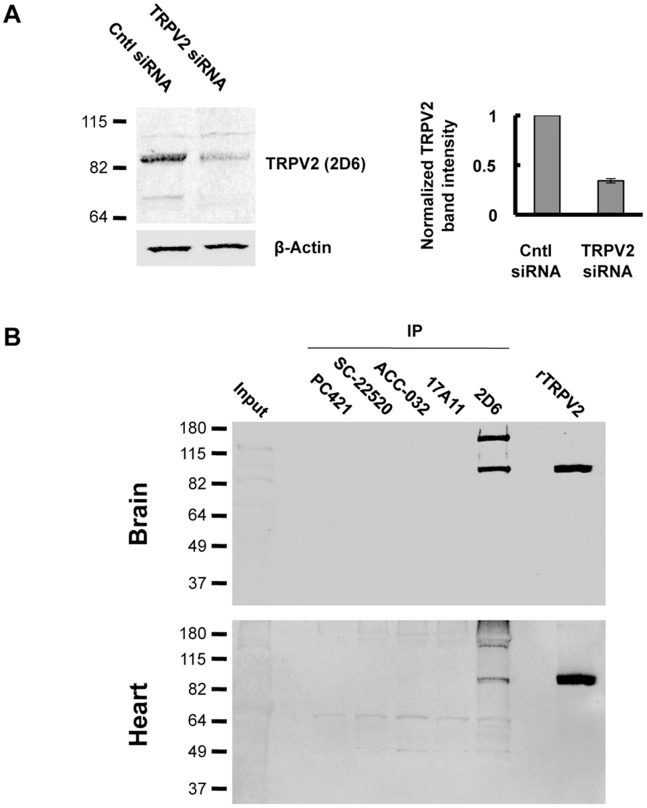
Detection of endogenous TRPV2. *A,* Western blot analysis with anti-TRPV2 2D6 of F11 cells treated with control siRNA or TRPV2 siRNA (100 nM, 48 h). Quantification of the band corresponding to the molecular weight of TRPV2 was measured using LiCor Odyssey software. TRPV2 band intensity was normalized to actin. Error bars represent S.E.M. from 3 separate experiments. *B,* Immunoprecipitation of TRPV2 with 10 µg of indicated antibodies from 2.5 mg mouse brain lysate and 2.5 mg mouse heart lysate. TRPV2 was detected by western blot with IR dye-labeled 2D6 antibody. Input represents 100 µg of total protein. Membranes from yeast overexpressing recombinant rat TRPV2 were loaded as a control.

An important test to validate antibody recognition of endogenously expressed protein is to determine specificity against tissues or cells in which the target protein has been reduced or deleted. F11 cells are derived from dorsal root ganglion (DRG) sensory neurons and provide a native-like environment for the study of thermoTRPV channels [21]. RT-PCR analysis demonstrated that both rat and mouse TRPV2 mRNA are present in F11 cells [24]. To determine recognition of endogenous TRPV2, F11 cells were transfected with siRNA to silence TRPV2 expression. Using 2D6, we observed a nearly 3 fold reduction in band intensity at the molecular weight corresponding to TRPV2 in cells treated with TRPV2 siRNA compared non-targeting siRNA (Figure 2A). ACC-032 recognized numerous bands that were not sensitive to TRPV2 siRNA, while SC-22520, PC421 and 17A11 showed no immunoreactivity with F11 cell extracts (data not shown). Out of the antibodies we tested, 2D6 is the best suited for detection of endogenous TRPV2 by western blot.

### Immunoprecipitation of TRPV2 from Mouse Brain and Heart

Determination of endogenous TRPV2 binding partners will aid in understanding the cellular function of the channel. An essential tool for identifying TRPV2 binding partners is an antibody suitable for immunoprecipitation of endogenous TRPV2. Based on quantitative studies of TRP channel mRNA levels in mouse organs, TRPV2 transcript was determined to be present in the highest amounts in the DRG, brain, and heart [25]. Therefore, we tested the efficiency of our monoclonal antibodies and the commercially available polyclonal TRPV2 antibodies for immunoprecipitation of endogenous TRPV2 from mouse brain and heart lysates. We found very little TRPV2 immunoreactivity in whole tissue extracts, suggesting that endogenously expressed TRPV2 protein is present in these tissues at very low levels (Figure 2B). The commercially available polyclonal antibodies, in addition to 17A11, failed to precipitate TRPV2 from mouse brain and heart (Figure 2B). 2D6 was able to precipitate TRPV2 from mouse brain and heart (Figure 2B). 2D6 antibody detected a higher molecular weight band that may correspond to TRPV2 dimer. These results indicate that 2D6 antibody is best suited for immunoprecipitation of TRPV2 and will likely provide a useful tool for determining physiological TRPV2 binding partners. Additionally, co-immunoprecipitation results obtained for endogenously expressed TRPV2 with the polyclonal antibodies we tested should be interpreted with caution.

### Detection of TRPV2 by Immunocytochemistry

**Figure 3:**
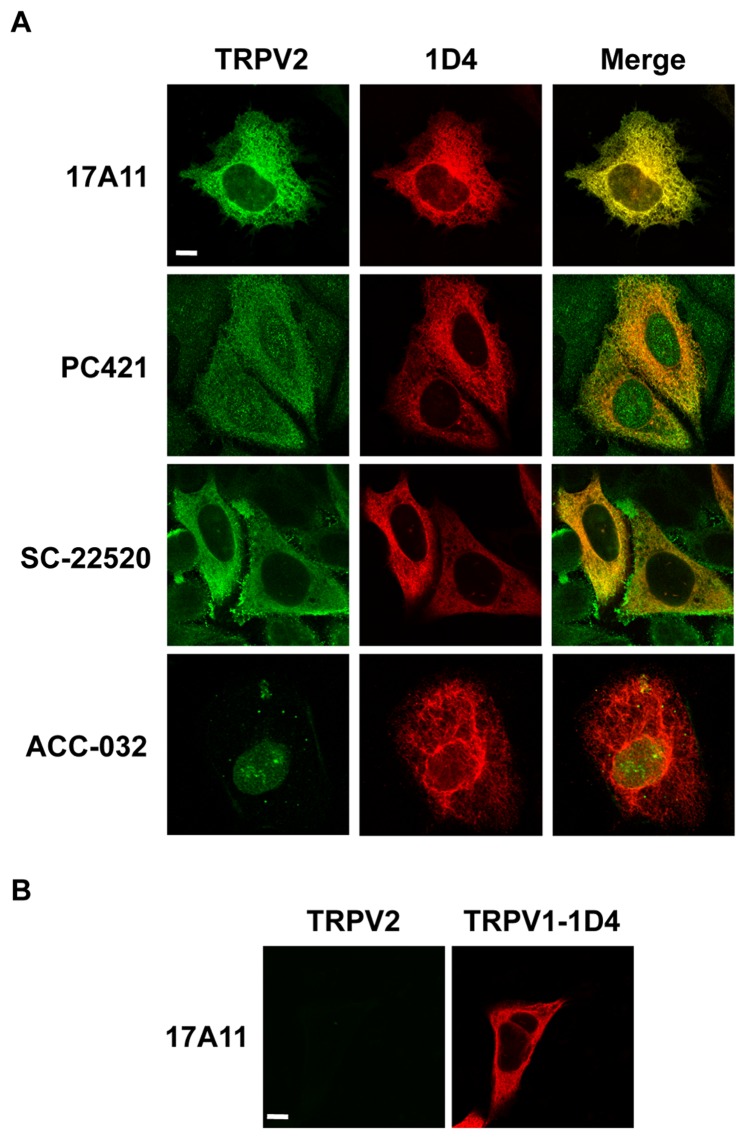
Immunostaining with TRPV2 antibodies. *A,* HeLa cells transiently expressing TRPV2-1D4 immunolabeled with indicated TRPV2 antibodies (green) and 1D4 antibody (red). Scale bar represents 10 µm. *B,* HeLa cells transiently expressing TRPV1-1D4 immunolabeled with TRPV2 17A11 (green) and 1D4 antibody (red). Scale bar represents 10 µm.

Polyclonal antibodies have been utilized to determine the factors that regulate trafficking of heterologously and endogenously expressed TRPV2 [7]. Therefore, employing properly validated antibodies to study the subcellular distribution of TRPV2 will be extremely important for understanding TRPV2 function in the cell. We tested the monoclonal antibodies generated in our laboratory as well as the commercially available polyclonal antibodies for recognition of TRPV2 by immunocytochemistry. To confirm the localization of heterologously expressed TRPV2, we transiently expressed 1D4-tagged TRPV2 in HeLa cells. 1D4 monoclonal antibody specifically recognizes the 1D4 epitope, allowing for certainty that 1D4 immunoreactivity corresponds to TRPV2 [26]. We found that 17A11 reacted specifically with 1D4 positive cells and co-localized with 1D4 at the subcellular level (Figure 3A). Additionally, 17A11 showed no cross-reactivity with 1D4-tagged TRPV1 (Figure 3B). 2D6 showed little immunofluorescence (data not shown). The commercially available polyclonal antibodies displayed immunoreactivity with both 1D4 positive and negative cells, and only partially co-localized with 1D4 (Figure 3A). PC421 showed limited immunofluorescence and did not co-localize with 1D4 at the subcellular level. Fluorescence corresponding to SC-22520 partially co-localized with 1D4 at the cell interior but also appears at the plasma membrane, whereas 1D4 fluorescence is only present in intracellular membranes. ACC-032 clearly did not co-localize with 1D4 fluorescence at the subcellular level. Our results indicate that 17A11 is the best candidate for specific recognition of endogenously expressed TRPV2.

### Effect of IGF-1 on Cell Surface Expression of TRPV2

Under basal conditions, TRPV2 localizes to the endoplasmic reticulum (ER); upon exposure to growth factors and mechanical stimulation, it is thought that TRPV2 translocates to the plasma membrane [7], [14]. In addition, increased surface expression of TRPV2 has been observed and implicated in several diseases, including muscular dystrophy, cardiomyopathy, and cancer [14], [16]. Since TRPV2 shows activity in the absence of ligand, the subcellular localization of the channel is likely to be important for its function [9].

**Figure 4:**
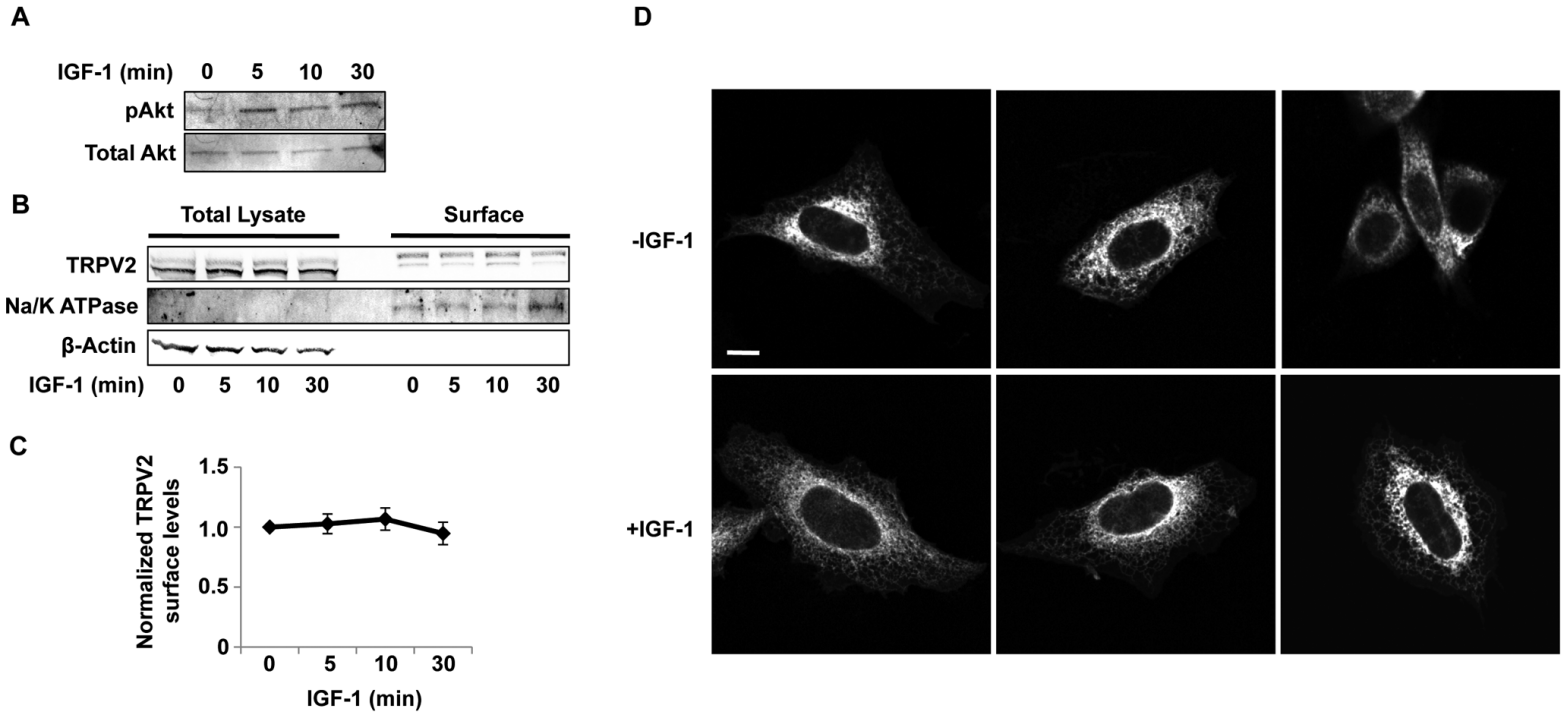
Regulation of TRPV2 trafficking by insulin-like growth factor-1. *A,* CHO-K1 cells were treated with IGF-1 (20 ng/ml) for the indicated times and immunoblotted with a phospho-Akt specific antibody. Membranes were then stripped and re-probed with a pan-Akt antibody. *B,* CHO-K1 cells transiently expressing TRPV2 were treated with IGF-1 (20 ng/ml) for indicated times. Surface proteins were biotinylated in intact cells at 37°C. Cells were then lysed and biotinylated proteins were captured with streptavidin agarose. Captured proteins were resolved by SDS-PAGE and detected by western blot with the indicated antibodies. Surface proteins represent the biotinylated fraction and the total lysate represents 5% of total protein. *C,* TRPV2 band intensity of the biotinylated fraction was measured using LiCor Odyssey software. Intensities were normalized to biotinylated Na/K ATPase band intensities. Error bars represent S.E.M. from 3 separate experiments. Differences are not statistically significant. *D,* CHO-K1 cells transiently expressing TRPV2 treated with vehicle (PBS) or IGF-1 (20 ng/ml) for 20 min were fixed and immunolabeled for TRPV2 (17A11 antibody). Images are representative of 3 separate experiments. Scale bar represents 10 µm.

**Figure 5:**
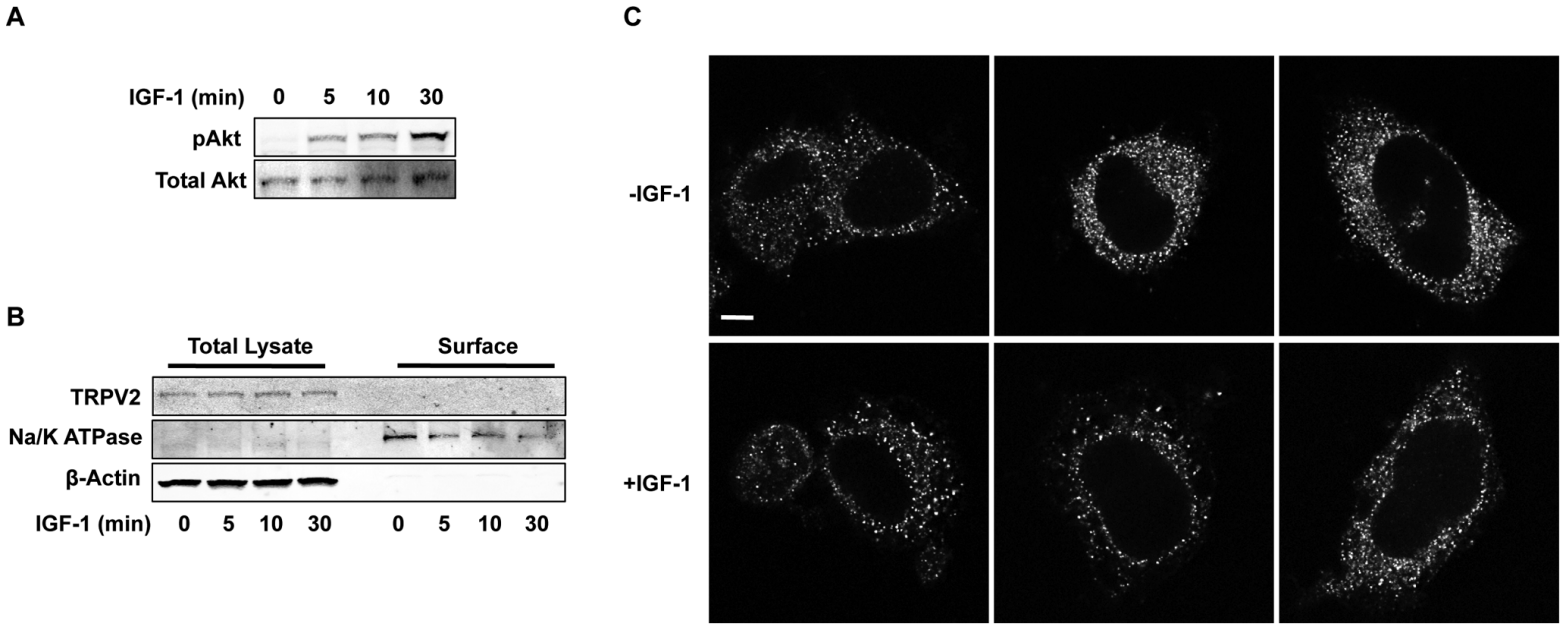
Effect of IGF-1 on endogenous TRPV2 trafficking in F11 cells. *A,* F11 cells were treated with IGF-1 (20 ng/ml) for the indicated times and immunoblotted with a phospho-Akt specific antibody. Membranes were then stripped and re-probed with a pan-Akt antibody. *B,* Biotinylation of surface proteins from F11 cells was performed following the procedure from Figure 5B. *C,* F11 cells treated with vehicle (PBS) or IGF-1 (20 ng/ml) for 20 min were fixed and immunolabeled for TRPV2 (17A11 antibody). Images are representative of 3 separate experiments. Scale bar represents 10 µm.

We employed our newly developed and characterized TRPV2 monoclonal antibodies to test the effects of IGF-1 on the subcellular distribution of TRPV2 transiently expressed in CHO cells as in the original studies of TRPV2 translocation [7]. Treatment of CHO cells with IGF-1 increased phospho-Akt levels indicating activation of the IGF-1 signaling pathway (Figure 4A). Cell surface biotinylation assays revealed a limited amount of heterologously expressed TRPV2 resides on the plasma membrane of CHO cells; however no significant changes in plasma membrane levels of TRPV2 were detected after exposure to IGF-1 for up to 30 minutes (Figure 4B, C). Two bands were detected with 2D6 antibody in the biotinylated fraction (Figure 4B). The higher molecular weight band likely corresponds to a TRPV2 glycosylation product. Additionally, immunocytochemistry revealed that most TRPV2 resides in an intracellular compartment, likely the ER, in both the absence and presence of IGF-1 (Figure 4D). We also tested the effects of IGF-1 on endogenous TRPV2 localization in F11 cells. IGF-1 treatment increased phospho-Akt levels in F11 cells (Figure 5A). Cell surface biotinylation and immunocytochemistry revealed that little to no TRPV2 is present at the cell surface in the absence and presence of IGF-1 (Figure 5B, C). Overall, these results are consistent with previous reports suggesting that while growth factor signaling may affect TRPV2 activity, it does not affect its plasma membrane expression [8], [9], [27].

## Discussion

Progress in understanding the cellular function of TRPV2 has been slowed by the lack of tools available to study the channel [12]. Studies of TRPV2 knockout mice have revealed that while TRPV2 may not have a major role in sensory transduction, it may perform some other important function [11]. Surface expression of TRPV2 was originally shown to be modulated by growth factors [7]. Additionally, increased plasma membrane expression of the channel has been implicated in the pathophysiology of a number of diseases [14], [16]. Regulated trafficking of TRPV2 to the cell surface remains controversial, as groups have employed different tools for studying channel translocation [12].

Conflicts in understanding TRPV2 trafficking may be based in the fact that TRPV2 antibodies raised against linear peptides were not suitable for immuno-detection of TRPV2. In order to study trafficking mechanisms of endogenously expressed TRPV2, we extensively characterized the commonly used commercially available TRPV2 polyclonal antibodies to determine if they were suitable for this goal. The antibodies we screened were PC421 (Calbiochem), SC-22520 (Santa Cruz) and ACC-032 (Alomone). PC421 is a commonly used antibody in TRPV2 studies [24], [28]. It was generated against peptide derived from the distal C-terminus of mouse and rat TRPV2 (KNSASEEDHLPLQVLQSP). This was the same epitope that was employed in the studies of TRPV2 identification [6]. We found that PC421 recognizes recombinant TRPV2 by western blot but it was not able to detect endogenous TRPV2 from F11 cells. Additionally, it failed to immunoprecipitate TRPV2 from mouse brain and heart lysates. Importantly, PC421 showed limited reactivity with TRPV2 by immunocytochemistry and possible cross-reactivity with another protein in the cell.

SC-22520 and other TRPV2 antibodies from Santa Cruz are also frequently utilized and were generated against an N-terminal peptide from human TRPV2 [17], [29], [30], [31]. SC-22520 did not detect full-length recombinant or endogenous TRPV2 by western blot and failed to immunoprecipitate TRPV2 from mouse tissues. Like PC421, SC-22520 also showed limited detection of TRPV2 by immunocytochemistry and appeared to cross-react with an unknown plasma membrane protein.

ACC-032, like PC421, was generated against a linear epitope from the rodent TRPV2 distal C-terminus (KKNPTSKPGKNSASEE). This was the same epitope used to generate antibodies that were employed to identify TRPV2 as a growth factor sensitive channel [7]. ACC-032 was able to detect full-length recombinant TRPV2 by western blot. However it detected multiple bands from F11 cell lysates that were not sensitive to TRPV2 siRNA. Additionally, it was not able to immunoprecipitate TRPV2 from mouse tissues and did not co-localize with TRPV2 at the subcellular level by immunofluorescence.

To overcome the difficulties and controversies in studying TRPV2 regulation, we generated monoclonal antibodies against full-length tetrameric TRPV2. After extensive screening, we found two monoclonal antibodies fit for detection of endogenously expressed TRPV2 (2D6 and 17A11). Both 2D6 and 17A11 were able to recognize recombinant full-length TRPV2 by western blot. 2D6 was the best candidate for western blot detection as it showed robust detection of heterologously expressed TRPV2 from HeLa cells and no cross-reactivity with the highly homologous TRPV1. Importantly, when endogenous TRPV2 expression was reduced in a neuronal cell line with siRNA, detection of TRPV2 with 2D6 was also diminished. Additionally, 2D6 was the only antibody we tested able to immunoprecipitate TRPV2 from mouse tissues. 17A11 was best suited for detection of TRPV2 by immunocytochemistry. 17A11 reacted specifically with cells expressing 1D4-tagged TRPV2 and co-localized with 1D4 immunofluorescence at the subcellular level. Moreover, this antibody, for the first time, was able to reveal the endogenous localization of TRPV2 in a neuronal cell line. Our analyses indicated that 2D6 is the best available antibody for detection of TRPV2 by western blot and immunoprecipitation while 17A11 is most suited for immunocytochemistry.

Much of the work to delineate the function of TRP channels relies on commercially available antibodies. Many of these antibodies are of poor quality and therefore have led to controversial and erroneous results [2]. An extensive characterization of these antibodies is needed in order to determine their efficacy in detection of specific TRP channels. Additionally, our approach using tetrameric TRPV2 as antigen may be applicable for generating antibodies against other TRP channels of unclear function.

It has been shown previously that IGF-1 regulates heterologously overexpressed and endogenous TRPV2 trafficking to the plasma membrane [7]. Although there is agreement that PI3 kinase signaling modulates TRPV2 activity, its effects on regulated insertion of TRPV2 into the plasma membrane remains controversial [8], [9], [27]. Since trafficking of TRPV2 appears to be important for its function, we used our newly validated TRPV2 monoclonal antibodies to test the hypothesis that growth factors, specifically IGF-1, increase surface expression of the channel.

Both cell surface biotinylation assays and immunocytochemistry indicated that IGF-1 had no effect on TRPV2 surface expression when transiently expressed in CHO-K1 cells. This is in direct contradiction with the original report that IGF-1 increases TRPV2 surface expression but in agreement with later studies showing that growth factor signaling only affects channel activity and not its plasma membrane levels [7], [8], [9]. Furthermore, we tested the effect of IGF-1 on plasma membrane expression of endogenous TRPV2 in DRG-derived F11 cells. Likewise, we found that little to no TRPV2 was present on the cell surface in the absence or presence of IGF-1.

Overall, our results indicate that TRPV2 primarily resides in intracellular membranes and its subcellular distribution is not sensitive to IGF-1 treatment. It will be important to use these newly characterized antibodies to determine if the expression and distribution of TRPV2 changes in disease states such as muscular dystrophy, cardiomyopathy and cancer.
